# Fine mapping of copy number variations on two cattle genome assemblies using high density SNP array

**DOI:** 10.1186/1471-2164-13-376

**Published:** 2012-08-06

**Authors:** Yali Hou, Derek M Bickhart, Miranda L Hvinden, Congjun Li, Jiuzhou Song, Didier A Boichard, Sébastien Fritz, André Eggen, Sue DeNise, George R Wiggans, Tad S Sonstegard, Curtis P Van Tassell, George E Liu

**Affiliations:** 1 Bovine Functional Genomics Laboratory, ANRI, USDA-ARS, BARC-East, Beltsville, MD 20705, USA; 2 Laboratory of Disease Genomics and Individualized Medicine, Beijing Institute of Genomics, Chinese Academy of Sciences, Beijing 100029, China; 3 Pfizer Animal Genetics, 333 Portage Road, Building 300, Kalamazoo, MI 49007, USA; 4 Department of Animal and Avian Sciences, University of Maryland, College Park, Maryland 20742, USA; 5 INRA UMR1313 Animal Genetics and Integrative Biology, Jouy-en-Josas 78352, France; 6 UNCEIA Genetics Team, Paris, 75595, France; 7 Illumina Inc., 5200 Illumina Way, San Diego, CA 92122, USA; 8 Animal Improvement Programs Laboratory, ANRI, USDA-ARS, Beltsville, Maryland 20705, USA

**Keywords:** Cattle genome, Breed, Copy number variation (CNV), Single nucleotide polymorphism (SNP)

## Abstract

**Background:**

Btau_4.0 and UMD3.1 are two distinct cattle reference genome assemblies. In our previous study using the low density BovineSNP50 array, we reported a copy number variation (CNV) analysis on Btau_4.0 with 521 animals of 21 cattle breeds, yielding 682 CNV regions with a total length of 139.8 megabases.

**Results:**

In this study using the high density BovineHD SNP array, we performed high resolution CNV analyses on both Btau_4.0 and UMD3.1 with 674 animals of 27 cattle breeds. We first compared CNV results derived from these two different SNP array platforms on Btau_4.0. With two thirds of the animals shared between studies, on Btau_4.0 we identified 3,346 candidate CNV regions representing 142.7 megabases (~4.70%) of the genome. With a similar total length but 5 times more event counts, the average CNVR length of current Btau_4.0 dataset is significantly shorter than the previous one (42.7 kb vs. 205 kb). Although subsets of these two results overlapped, 64% (91.6 megabases) of current dataset was not present in the previous study. We also performed similar analyses on UMD3.1 using these BovineHD SNP array results. Approximately 50% more and 20% longer CNVs were called on UMD3.1 as compared to those on Btau_4.0. However, a comparable result of CNVRs (3,438 regions with a total length 146.9 megabases) was obtained. We suspect that these results are due to the UMD3.1 assembly's efforts of placing unplaced contigs and removing unmerged alleles. Selected CNVs were further experimentally validated, achieving a 73% PCR validation rate, which is considerably higher than the previous validation rate. About 20-45% of CNV regions overlapped with cattle RefSeq genes and Ensembl genes. Panther and IPA analyses indicated that these genes provide a wide spectrum of biological processes involving immune system, lipid metabolism, cell, organism and system development.

**Conclusion:**

We present a comprehensive result of cattle CNVs at a higher resolution and sensitivity. We identified over 3,000 candidate CNV regions on both Btau_4.0 and UMD3.1, further compared current datasets with previous results, and examined the impacts of genome assemblies on CNV calling.

## Background

Genomic structural variation including copy number variation (CNV) has been intensively studied in human [1-4] and rodents [5-8]. Dozens of human and mouse CNV studies have demonstrated that some CNVs are associated with phenotypic traits and diseases [9-12]. Initial CNV reports also appeared in domesticated animals, including dog [13-15], cattle [16], chicken [17,18], pig [19,20], sheep, and goat [21,22]. Recent bovine CNV studies have generated several cattle CNV maps using various approaches [23-26]. In our previous study, we performed an analysis of CNV using the Bovine HapMap SNP genotyping data, including 539 animals of 21 modern cattle breeds and 6 outgroups [27]. Efforts to explore the association between cattle CNV and economical traits have been published [28,29], even though the actual functional mechanisms are not yet well defined.

CNV can be identified using various approaches, including comparative genomic hybridization (CGH) array, SNP array, and next-generation sequencing. Compared to other approaches, the advantages of SNP array include its low cost, dense coverage, and high throughput. Substantial genotyping data have been produced from genome-wide association studies, which can be directly exploited for the CNV analysis. A wide range of algorithms of CNV discovery based on SNP array has been developed, including CNVPartition, QuantiSNP [30], PennCNV [31], Birdsuite [32], Cokgen [33], and others. Reviews of the strengths and weaknesses of these algorithms have been published [34,35]. As one of the leading methods, PennCNV incorporates multiple sources of information, including total signal intensity and allelic intensity ratio at each SNP marker, the distance between neighboring SNPs, and the allele frequency of SNPs. PennCNV also integrates a computational approach by fitting regression models with GC content to overcome "genomic waves" [36,37]. Furthermore, PennCNV is capable of considering pedigree information (parent-offspring trios) to improve call rates and accuracy of breakpoint prediction as well as to infer chromosome-specific SNP genotypes in CNVs.

The availability of two alternative cattle reference genomes (Btau_4.0 and UMD3, [38,39]) has opened new avenues of cattle genome research. With the advent of next-generation sequencing, more high density SNP arrays were made commercially available, including Illumina BovineHD BeadChip with more than 750,000 SNPs (Van Tassell et al., unpublished; [40]), which is 15-fold denser as compared to the previous BovineSNP50 array. Furthermore, then-published CNV results were incorporated during the BovineHD design phase to increase its coverage in variable genomic regions.

Based on this high density BovineHD SNP array, our goals in this study were to perform high resolution CNV analyses on both Btau_4.0 and UMD3.1, to compare them with the previous results, and to examine the impacts of genome assemblies on CNV calling.

## Results and Discussion

### Cattle CNV identification

As described previously [27], we performed CNV calling on both Btau_4.0 and UMD3.1 assemblies. Due to mapping uncertainty, we excluded chrX and chrUn from our analysis.

**Figure 1:**
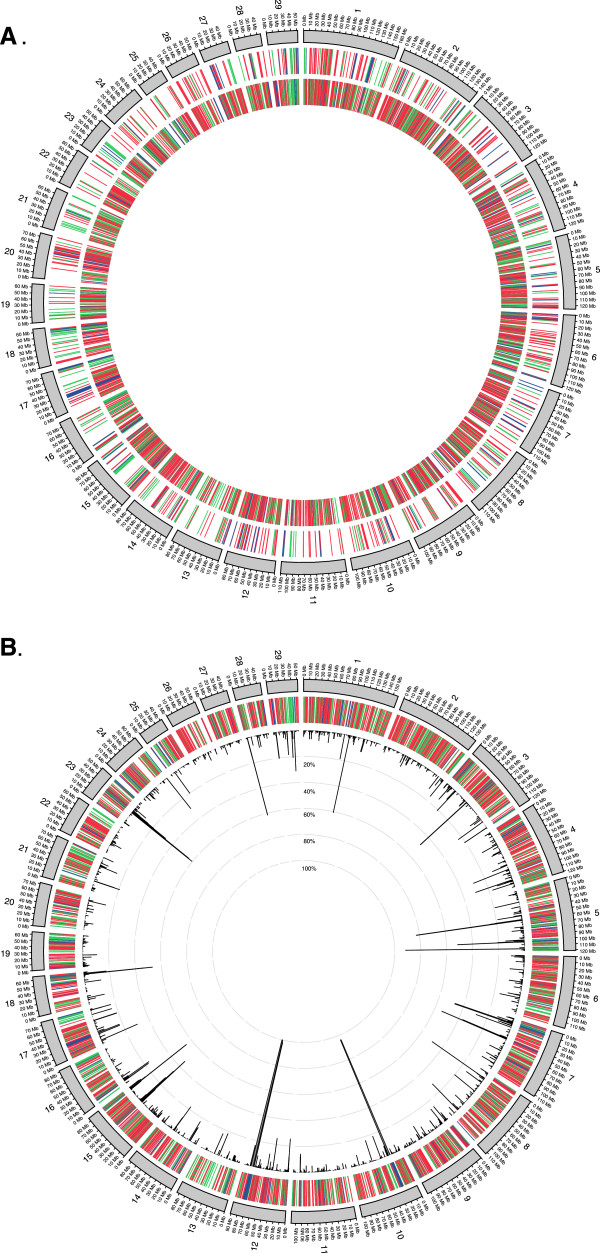
**Cattle copy number variations derived from SNP arrays on Btau_4.0 and UMD3.1. A.** On Btau_4.0, CNV regions (682 events, 139.8 Mb) derived from BovineSNP50 assay are shown in the outer circle in green (gain), red (loss) and dark blue (both), while the inner circle shows the CNV regions (3,346 events, 142.7 Mb) derived from BovineHD assay. **B**. On UMD3.1, CNV regions (3,438 events, 146.9 Mb) derived from BovineHD assay are shown in the outer circle in green (gain), red (loss) and dark blue (both), while the inner circle shows their frequencies.

On the Btau_4.0 assembly, 34,311 CNVs were detected with an average of 51 events for each animal (Table 1, Additional file 1: Table S2, Figure 1A and Additional file 2: Figure S1). The average CNV length was 39,953 bp. For subspecies/groups such as the Taurine, Indicine, Composite (Taurine × Indicine) and African breeds, the average CNV events per sample were 45, 65, 53 and 66 respectively (i.e. T:I:C:A = 45:65:53:66). Indicine and African breeds had the most CNVs identified. Within each subspecies/group, the numbers of unique CNVs ranged from 2.3 to 5.1 per sample, indicating that the majority of CNVs were shared at least by two individuals within the same subspecies/groups.

**Table 1:** CNVs or CNVRs on Btau_4.0 and UMD3.1

***Subspecies/groups***	***Sample***	***Count***	***Unique***	***Gain***	***Loss***	***Total Length***
**Btau_4.0**						
***Taurine***	447	20,302 (45.4)	1,044 (2.3)	7,916 (17.7)	12,386 (27.7)	814,447,018 (40,117)
***Indicine***	113	7,352 (65.1)	309 (2.7)	2,595 (23.0)	4,757 (42.1)	266,853,291 (36,297)
***Taurine × Indicine***	67	3,569 (53.3)	198 (3.0)	1,508 (22.5)	2,061 (30.8)	159,735,770 (44,756)
***African Breeds***	47	3,088 (65.7)	240 (5.1)	1,248 (26.6)	1,840 (39.1)	129,777,675 (42,026)
**Total**	674	34,311 (50.9)	1,791 (2.7)	13,267 (19.7)	21,044 (31.2)	1,370,813,754 (39,953)
**CNVR**	674	3,346 ^a^	1,316 ^b^	986 ^c^	2,051 ^c^	142,718,107 (42,653)
**UMD3.1**						
***Taurine***	434	32,445 (74.8)	1,052 (2.4)	12,419 (28.6)	20,026 (46.1)	1,551,624,380 (47,823)
***Indicine***	97	8,715 (89.8)	327 (3.4)	3,102 (32.0)	5,613 (57.9)	394,966,292 (45,320)
***Taurine × Indicine***	63	5,332 (84.6)	237 (3.8)	2,268 (36.0)	3,064 (48.6)	256,749,182 (48,153)
***African Breeds***	36	3,212 (89.2)	230 (6.4)	1,197 (33.3)	2,015 (56.0)	151,559,764 (47,185)
**Total**	630	49,704 (78.9)	1,846 (2.9)	18,986 (30.1)	30,718 (48.8)	2,354,899,618 (47,378)
**CNVR**	630	3,438 ^a^	1,360 ^d^	1,054 ^e^	2,042 ^e^	146,905,950 (42,730)

Numbers in parentheses are values normalized by sample counts, except in the case of the parentheses values in the "total length" column, which are average lengths normalized by CNV counts. ^a ^These numbers represent nonredundent CNVR counts. In Btau_4.0, ^b ^1,316 out of 3,346 CNVRs were unique to one sample, 2,030 CNVRs were shared by at least 2 individuals and 179 of 2,030 multiple events had a frequency > =5%. ^c ^Besides the 986 gain and 2,051 loss CNVRs, there were 309 CNVRs containing both loss and gain events. In UMD3.1, ^d ^1,360 out of 3,438 CNVRs were unique to one sample, 2,078 CNVRs were shared by at least 2 individuals and 230 of 2,078 multiple events had a frequency > =5%. ^e ^Besides the 1,054 gain and 2,042 loss CNVRs, there were 342 CNVRs containing both loss and gain events.

When we merged CNVs into nonredundent CNV regions (CNVRs), a total of 3,346 events were identified covering 142.7 Mb of polymorphic sequence, corresponding to 5.61% of the autosomal genome sequence (142.7/2,545.9 Mb) and 4.89% of the whole cattle genome (142.7/2,918.0 Mb, Table 1, Figure 1A and Additional file 3: Table S3). These CNVRs were comprised of 2,051 loss events, 986 gain and 309 both (loss and gain within the same region), ranging from 1,018 to 5,552,622 bp (Additional file 3: Table S3). Loss events are approximately 2.1-fold more common than gain events, but have smaller sizes than gain events on average (28.5 kb vs. 37.6 kb).

Furthermore, 1,316 CNVRs were found in only one sample (Unique), 2,030 CNVRs were shared at least by two animals (Multiple or more), and 179 events had a frequency >5% (Additional file 3: Table S3). These results suggest that segregating CNVs exist among these subspecies, breeds and groups, which is consistent with our earlier results [26,27].

Strikingly, the mean and median CNVR lengths were significantly shorter, 42,653 and 15,794 bp respectively, when compared to values derived from our previous low density SNP50 array study (mean: 204,965 and median: 131,179 bp). With a similar total length of ~140 Mb, our new dataset contains almost five times the number of detected CNVs (3,346) than our previous study (682), suggesting that the BovineHD array provides higher resolution and sensitivity for CNV discovery. Also the current CNV results seem to be more uniformly distributed, with more events detected in the centromere and telomere regions compared to the previous results (For example, chr10 and chr20 in Figure 1A).

We also made CNV calls using the UMD3.1 assembly (Figure 1B and Table 1). For UMD3.1, 630 animals passed the PennCNV quality filtering, 44 fewer as compared to Btau_4.0. When comparing results between Btau_4.0 and UMD3.1, more CNVs of longer length were found on UMD3.1 than those on Btau_4.0 (Additional file 1: Table S4 and Additional file 4: Table S5). However, the relative differences in CNV counts across distinct species/breeds were preserved (i.e. T:I:C:A ratio: 45:65:53:66 vs. 75:90:85:89) and the merged CNV region results (count and length) were consistent.

Compared to the Btau_4.0 results, 44.86% more CNVs (49,704) were detected within the placed autosomes and an average of 54.90% more events (79) were obtained for each sample on UMD3.1 (Additional file 1: Table S4). The average length of CNV was also 18.58% larger (47,378 bp) when using the UMD3.1 assembly coordinates; however, we identified a similar number of CNVRs (3,438 events) compared to Btau_4.0 CNVRs. The total lengths of CNVRs were similar with comparable statistics (146,905,950 bp; only 2.93% larger, Additional file 4: Table S5). When we assessed the difference in CNVR type calls (gain, loss and both), counts for each category varied less than 70 between the Btau_4.0 and UMD3.1 assemblies.

These results were not unexpected in light of the attributes of these two assemblies. As both assemblies are based on the same raw whole-genome shotgun reads, the most obvious difference between the two is that the Btau_4.0 unplaced contigs from chrUn are now placed on UMD3.1. Another difference between them is that local duplication artifacts, or duplicated regions that were artificially created on the Btau_4.0 assembly, were removed on UMD3.1. As our previous segmental duplication analyses detected, 267 Mb of duplicated sequence on Btau_4.0 were considered to be artifacts from a failure to merge high identity alleles on the reference assembly [41]. Our results were supported by independent FISH experiments [42] and similar observations reported by Zimin et al [39]. In summary, UMD3.1 seems to be more favorable than Btau_4.0 in terms of placing unplaced contigs and removing the unmerged alleles. As a result, most of CNVs calls derived from chrUn of Btau_4.0 could be recovered in UMD3.1 results. It is interesting to note even though UMD3.1’s CNV calls are more abundant and larger, the merged CNVR results were almost equivalent in terms of count and length. However, the exact effects of local assembly differences (besides UMD3.1’s placing unplaced contigs and removing the unmerged alleles) on CNV calling (count and length) warrant more detailed investigations in the future.

### Quality assessment of selected CNV Events

**Figure 2:**
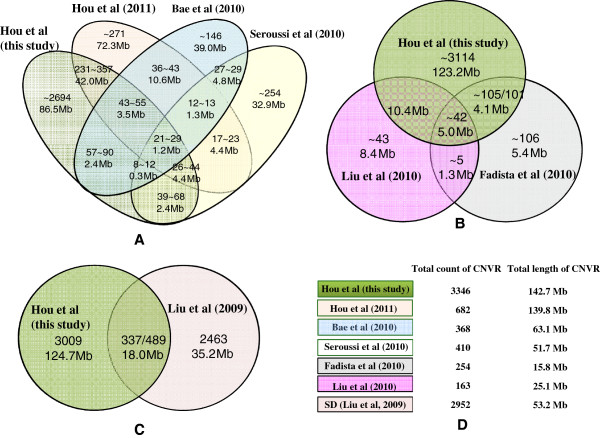
**Comparisons between 3,346 autosomal CNVRs identified in this study and the other cattle CNVR datasets and the segmental duplication (SD) dataset on Btau_4.0. A**, three CNVR datasets derived from SNP array (Hou et al., 2011, [24,25,27]); **B**, two CNVR datasets derived from array CGH studies [23,26]; **C**, the SD dataset [41]; **D**, the summaries and legends of existing cattle CNVR and SD datasets.

Since most existing cattle CNV studies were based on Btau_4.0, we first compared the identified CNV regions with previous published CNVs dataset [23-26] and the segment duplication (SD) dataset [41] based on Btau_4.0 (Figure 2). Since calls on chrX were not always included in these studies, we only compared the CNVRs detected within the autosomes. We found that 789 of our 3346 CNVRs (65.1 Mb) overlapped with all combined nonredundant published data. Detailed information of each comparison was displayed in Figure 2. Approximately 14% of our new CNV calls (482 out of 3346 CNVRs) overlapped with 51% (346 out of total 682 CNVR) of the CNV regions identified in 521 animals of 21 cattle breeds using BovineSNP50 arrays [27]. When considering the CNVR lengths, 36% of variable sequence space identified in this study intersected with 37% of the previous study, representing an overlap of 51.1 Mb. The discrepancy between these two studies could be due to a difference in samples as 674 animals of 27 cattle breeds were used in this study out of which, approximately 442 animals of 19 breeds (~65.6%) were shared by the later study. Additionally, SNP arrays that probed 763,572 SNPs were used in this study, while arrays with only 56,947 SNPs were used in the previous study. Only 46,475 SNPs were shared by both platforms.

After comparison with other existing datasets, we found that 54.38% (77.6 Mb) of our CNVR calls were not reported in the literature. In order to confirm these novel CNVRs, we performed 56 quantitative PCR (qPCR) assays for 18 CNV loci in 12 animals (Additional file 5: Table S6). All primer coordinates on both assemblies were given in Additional file 5: Table S6, with the exception that primers for CNVR No. 1856 could only be placed on the Btau_4.0 assembly. Most of the CNV regions had two target amplicons placed near the probes in the CNVR regions. Out of 28 CNV loci and animal combinations, 23 (82.14%) had positive qPCR confirmations in at least one amplicon and 18 (64.30%) had positive results at both amplicons. If each PCR assay was counted separately, 73.21% (41/56) were in an agreement with the CNV status estimated by PennCNV (Additional file 5: Table S6), which is considerably higher than the previous validation rates between 48 to 60% [27].

### CNVs overlap with segmental duplications and other genomic features

We also compared the identified CNV regions with the 2,952 SD (excluding chrX and chrUn) identified by WGAC and WSSD [41]. Agreeing with previous predictions regarding cattle SDs, a local tandem distribution pattern is predominant in our cattle CNVR dataset (Figure 1A). About 10.07% (337/3346) of CNV regions directly overlap with cattle SDs with an overlapping span of 18 Mb (12.61% of the total 142.7 Mb). Approximately 16.57% (489/2952) of the SDs (excluding chrX and chrUn) identified by WGAC and WSSD exhibit CNVs (Figure 2C). In comparison, 25.66% of the CNVRs (175/682) detected by using BovineSNP50 array overlap with cattle SDs, corresponding to 16.28 Mb (11.65% of the total 139.8 Mb) [27]. The fractions of CNVR calls that overlap with SDs were similar (12.61% vs. 11.65%) in both SNP array studies and they were significantly lower than the fraction of SD-overlapping CNVRs (58.90%, 96/163 or 60.56%, 15.2/25.1 Mb) as detected by using array CGH [26]. This lower overlap fraction probably reflects the fact that both SNP arrays have poor representation within cattle SD regions. SNP density on the BovineSNP50 array drops by one-third (from 21 probes/Mb in unique regions down to 14 probes/Mb) in SD regions while SNP density on the BovineHD array drops by over 61% (from 277 probes/Mb in unique regions down to 107 probes/Mb) in SD regions. We also overlapped our CNVRs with cattle Online Mendelian Inheritance in Animals (OMIA), Online Mendelian Inheritance in Man (OMIM) and cattle QTL datasets (Additional file 3: Table S3).

### Gene content of CNV regions

Since UMD3.1 placed unplaced contigs and removed unmerged alleles, we focused on further characterization of the 3,438 high-confidence CNV regions from UMD3.1 autosomes. Additionally, we used both Btau_4.0 and UMD3.1 assemblies and obtained similar Panther and IPA results, as presented in Additional file 6: Table S7 and Additional file 7: Table S8.

We investigated the gene content of CNV regions derived from both Btau_4.0 and UMD3.1 (Table 2). Within the 3,438 CNVRs on UMD3.1, there were 939 unique cattle RefSeq genes and 2,153 Ensembl peptides, corresponding to 1,855 unique Ensembl genes (Table 2 and Additional file 4: Table S5). Approximately, 24% (817/3,438) of our CNVRs spanned cattle Refseq genes and 34% (1,165/3,438) of them overlapped with Ensembl genes. A detailed list of CNVR-gene overlapping is displayed in Additional file 4: Table S5. Beside those reported before such as *ABCC4*, *ATP8A1*, *IGLL1*, LYZ, *PGR*, *SGCD*, *SCP2*, WC1, ULBP, OR, *ZNF280A*, *HLA-DQA* and *BLA-DQ*, novel CNVR related genes or gene families include *GBP6*, *SCMH1*, *GIMAP7*, *UGT2B10*, *ORM1* and others.

**Table 2:** Gene contents of CNVRs on Btau_4.0 and UMD3.1

	**Cattle RefSeq**	***in silico *mapped human RefSeq**	**Ensembl genes**	**Glean genes**
**Btau_4.0**				
#genes	935 (836)	5,549 (3,146)	1,890 (1,788)	2,536 (2,408)
#overlapped CNVR	703 (21%)	1,507 (45%)	1,116 (33%)	1,330 (40%)
**UMD3.1**				
#genes	1,062 (939)	6,497 (3,828)	1,964 (1,855)	ND
#overlapped CNVR	817 (24%)	1,533 (45%)	1,165 (34%)	ND

Numbers in parentheses for #genes are the number of unique nonredundent gene counts, as one gene could show up multiple times in different CNVRs. Numbers in parentheses for #overlapped CNVR are the percentages of overlapped CNVR in the total CNVRs. ND: Glean genes on UMD3.1 were not determined.

We assigned PANTHER accessions to the overlapped peptides for CNVRs identified on both Btau_4.0 and UMD3.1 in this study. Similar statistically significant overrepresentations were observed for multiple categories (Additional file 6: Table S7). This set of copy number variable genes encompasses a wide spectrum of molecular functions, biological processes, pathways, cellular components and Panther protein classes. For example, immune system process, cellular defense response, response to stimulus, antigen processing and presentation, natural killer cell activation were among several enriched biological processes. G-protein coupled receptor activity was enriched while transcription factor and regulation activities were under represented in the Molecular Function terms.

Our UMD3.1 CNVRs overlapped with 3,828 unique *in silico* mapped human RefSeq genes (Table 2 and Additional file 4: Table S5), of which 3,669 were mapped as candidate genes for IPA analysis. A total of eleven networks with an IPA score greater than 10 were identified (Table 3). A score of 10 indicates that there was less than a 10^-10^ probability that the genes in the network were associated together by chance. The identified regulatory networks covered a broad range of physiological functions and processes, including inflammatory response, cell-to-cell signaling and interaction, cell development and cycle, lipid metabolism, normal development and function (embryo, organism, reproductive system, hematological system, skeletal and muscular system), and genetic disorder (Additional file 7: Table S8). Immunological and defense pathways were particularly enriched in IPA Pathway and Function analyses, further confirming the Panther results. The results of our functional analyses are in agreement with the findings of previous CNV studies [23-27]. Using the Btau_4.0 assembly, the IPA results are generally similar, with slight differences likely attributable to differences in assemblies and their annotations.

**Table 3:** Network analyses using IPA based on UMD3.1

**ID**	**Score**	**Focus Molecules**	**Top Functions**
1	28	31	Reproductive System Development and Function, Cellular Development, Cellular Growth and Proliferation
2	27	30	Inflammatory Response, Inflammatory Disease, Cell-To-Cell Signaling and Interaction
3	27	30	Lipid Metabolism, Molecular Transport, Small Molecule Biochemistry
4	27	30	Cellular Development, Cellular Growth and Proliferation, Decreased Levels of Albumin
5	25	29	Cellular Assembly and Organization, Cellular Function and Maintenance, Cancer
6	17	24	Gene Expression, DNA Replication, Recombination, and Repair, Cancer
7	11	17	DNA Replication, Recombination, and Repair, Cancer, Gastrointestinal Disease
8	11	19	Cell Cycle, Cancer, Genetic Disorder
9	11	17	Cellular Development, Hematological System Development and Function, Hematopoiesis
10	11	15	Cellular Growth and Proliferation, Tumor Morphology, Inflammatory Response
11	10	14	Embryonic Development, Organismal Development, Skeletal and Muscular System Development and Function

## Conclusion

In addition to single nucleotide polymorphisms (SNPs), CNVs have been revealed to be a substantial source of genetic variation in cattle. In this study, we performed two comprehensive CNV analyses based on Btau_4.0 and UMD3.1. When we compared our current and previous results across different SNP platforms on Btau_4.0, we detected higher resolution, sensitivity and PCR validation rate in these current CNV datasets. Our findings suggest that the use of high-density oligonucleotide arrays may allow more precise boundary information to be extracted for CNV detection. Therefore, the use of high-density SNP arrays combined with improved CNV calling algorithms seems to significantly improve the accuracy of CNV calling. As a consequence, more CNVs were identified and the CNVs identified from those SNP arrays were more accurate and with better defined boundaries.

In this study, we provide additional support to the observation that the distribution of CNVs varies among the four subspecies groups. In case of the complete absence of a particular allele of a SNP in a certain breed, the copy number of that SNP in the breed would be biased towards loss or deletion. While some of these breed differences could be related to the fact that the SNP markers were designed on the reference genome sequence (which was derived from the sequence of a Hereford cow of European origin; L1 Dominette 01449), our observation of differential CNV counts in different breeds and subspecies was largely consistent with their histories and divergences.

We also systematically evaluated current results across two existing cattle reference assemblies. Although we obtained comparable CNVR results, we found approximately 50% more and 20% longer CNVs on UMD3.1 as opposed to those on Btau_4.0. It is worth to note significant differences exist between different assemblies. Therefore it is critical to select appropriate assemblies and further validate the predicted variants experimentally.

## Methods

### Selection of cattle breeds and animals

Cattle selected in this study were composed of 686 individuals from 27 breeds originally, out of which, 674 distinct high quality genotyping results with call rate larger than 99.70% remained after PennCNV quality filtering. This panel included 447 animals from 18 taurine breeds, 113 animals from three breeds of predominantly indicine background, 67 animals from three breeds that are Taurine × Indicine Composites, and 47 animals from three African breeds (Additional file 1: Table S1). It is worth to note that for each subspecies, breeds or individuals within breeds were sampled from more than one continent to represent the global cattle population. This panel contained 41 trios where both parents and an offspring were genotyped.

### Identification of cattle CNVs

For research use, the Infinium BovineHD BeadChip array features 786,799 evenly spaced SNPs probes based on assembly UMD3.1 and out of which, 783,617 were placed on autosomes and the X chromosome. This multi-sample genotyping panel delivers >99% call rate and >99.9% reproducibility, along with the ability to detect CNV ( http://www.illumina.com/Documents//products/datasheets/datasheet_bovineHD.pdf). Since we also surveyed on Btau_4.0, SNP probe coordinates were migrated from UMD3.1.0 to Btau_4.0 by using both UCSC liftOver ( http://hgdownload.cse.ucsc.edu/admin/exe/) and Blat tools. Approximately 97% (763,572/783,617) of the probes on autosomes and the X chromosome were converted successfully. CNV was detected using the PennCNV algorithm as described previously [27]. PennCNV incorporates multiple sources of information together, including Log R Ratio (LRR) and B Allele Frequency (BAF) at each SNP marker for each individual, more realistic models for state transition between different copy number states based on the distance between neighboring SNPs, population frequency of B allele (PFB), the allele frequency of SNPs, and the pedigree information where available, into a hidden Markov model (HMM). Both LRR and BAF of each SNP for each individual were exported from Illumina GenomeStudio Genotyping Module v1.9 software using the default clustering file. The PFB file was calculated as the average BAF for each marker in this population. Genomic waves, calculated as the GC content of the 1 Mb genomic region surrounding each marker (500 kb each side), were corrected by performing the -gcmodel option. Pedigree information including 41 trios were used to improve the accuracy of CNV identification. As described previously [27], PennCNV algorithm (with options: -test or -joint) was applied to autosomes (with option: -lastchr 29) to detect cattle CNV. After detection, 674 out of 686 animals passed the standard filtering of low-quality samples with the default cutoffs (standard deviation of LRR as 0.30, BAF drift as 0.01, and waviness factor as 0.05). The final CNVs set was the nonredundant combination of CNVs from the -joint results for family trio members and the -test results for unrelated individuals. CNVRs were determined by aggregating overlapping CNVs identified across samples.

### qPCR validation

Primers were designed using Primer3 ( http://frodo.wi.mit.edu/primer3/) with a limitation of amplicon length to 150 bp to 250 bp, as well as CG clamp as 2. All other Primer3 settings were left at the default values. Primer information is shown in Additional file 5: Table S6. qPCR experiments were conducted using SYBR green chemistry in triplicate reactions, each with a reaction volume of 25 μl. All reactions were amplified on a BioRad MyIQ thermocycler. An intron-exon junction of the BTF3 gene was chosen as a reference location for all qPCR experiments. Analysis of resultant crossing thresholds (C_T_) was performed using the relative comparative C_T_ method. Calibrations of C_T_ values were derived from amplification of reference and test primers on a genomic DNA template derived from sequenced cow, L1 Dominette 01449, an European-origin Hereford. Since all reference and test primers did not overlap with any of Dominette’s CNV regions, two-copy states were assumed for both amplicons in Dominette. The copy number for each test region was calculated as 2^(1+dd^^CT)^.

### Gene content

Gene content of cattle CNV regions was assessed using cattle RefSeq and *in silico* mapped human RefSeq, the Glean consensus gene set (the UCSC Genome Browser website at http://genome.ucsc.edu/), Ensembl genes ( ftp://ftp.ensembl.org/pub/current_fasta/bos_taurus/pep/). We obtained a total of 26,977 and 22,118 bovine peptides from Ensembl on Btau_4.0 and UMD3.1 respectively. In addition, using the PANTHER classification system, we tested the hypothesis that the PANTHER biological process, molecular function, pathway, cellular component, and Panther protein class terms were under- or overrepresented in CNV regions after Bonferroni corrections [26]. It is worth noting that a portion of the genes in the bovine genome has not been annotated or has been annotated with unknown function, which may influence the outcome of this analysis. Overlapping between CNVRs and additional genomic features such as cattle OMIA, OMIM and cattle QTL datasets were performed as described [26].

*In silico* mapped human RefSeq genes in CNVRs were analyzed using Ingenuity Pathways Analysis (IPA) v9.0 (Ingenuity Systems, Redwood City, CA) as previously described [27]. The accessions of unique genes were imported into the software and subsequently mapped to their corresponding annotations in the Ingenuity Pathways Knowledge Base. The ''Core Analysis” function included in IPA ( http://www.ingenuity.com/) was used to analyze these genes in the context of networks, biological functions and Pathways. The networks accommodated these unique genes (also called focus molecules) were identified in comparison with the comprehensive global networks developed by IPA. The molecule network was illustrated with an assigned relevance score, the number of focus molecules, as well as the top function of the networks. In the process of analysis, each network was set to have a maximum of 35 molecules by default. We used only human genes and all confidence levels, including evidences of experimentally observed, predicted high or moderate confidence. The top significant biological functions and Pathway were listed.

## Abbreviations

aCGH: array comparative genomic hybridization; CNV: copy number variation; CNVR: CNV region; C_T_: threshold cycle; DT: Dominette; NGS: next-generation sequencing; qPCR: quantitative PCR; QTL: Quantitative trait locus; OMIA: Online Mendelian Inheritance in Animals; OMIM: Online Mendelian Inheritance in Man; SD: segmental duplication; SNP: single nucleotide polymorphism.

## Competing interests

M.L.H. and S.D. are employees of Pfizer Animal Genetics. A.E. is an employee of Illumina, Inc. The other authors declare that they have no competing interests.

## Author contributions

GEL and YH conceived and designed the experiments. DMB, YH, CL, and JS performed *in silico* prediction, qPCR, and computational analyses. MLH, DAB, SF, AE, SD, GRW, TSS and CPVT collected samples and generated SNP array genotyping data. YH, GEL, and DMB wrote the paper. All authors read and approved the final manuscript.

## Supplementary Material

Additional file 1 **Table S1. **Numbers of subspecies, breeds, animals and trios used to call CNVs genotyped by BovineHD assay**. Table S2. **The summary of CNVs or CNVRs for each specie/breed based on Btau_4.0. **Table S4. **The summary of CNVs or CNVRs for each specie/breed based on UMD3.1. Click here for file

Additional file 2 **Figure S1. **Comparison of cattle copy number variations derived from BovineHD and BovineSNP50 assays on Batu_4.0. Click here for file

Additional file 3 **Table S3. **Btau_4.0 CNV regions, their frequencies, corresponding gene contents, QTL, OMIM, and OMIA overlapping information. Click here for file

Additional file 4 **Table S5. **UMD3.1 CNV regions, their frequencies, corresponding gene contents. Click here for file

Additional file 5 **Table S6. **The summary of PCR results. Click here for file

Additional file 6 **Table S7. **Over/Underrepresentation of PANTHER terms (molecular function, biological process, pathway, cellular component and PANTHER protein class) on Batu_4.0 and UMD3.1. Click here for file

Additional file 7 **Table S8: **Network, Biological function and Pathway analyses using IPA on Batu_4.0 and UMD3.1. Click here for file
