# Supplementary material for: Fine mapping of copy number variations on two cattle genome assemblies using high density SNP array
Source: BMC Genomics. 2012 Aug 6;13:376. doi: 10.1186/1471-2164-13-376 (PMC3583728; doi:10.1186/1471-2164-13-376)
Supplement: Additional file 1 — Table S1. Numbers of subspecies, breeds, animals and trios used to call CNVs genotyped by BovineHD assay. Table S2. The summary of CNVs or CNVRs for each specie/breed based on Btau_4.0. Table S4. The summary of CNVs or CNVRs for each specie/breed based on UMD3.1. [file 1471-2164-13-376-S1.doc]

**Table S1. Numbers of subspecies, breeds, animals and trios used to call CNVs genotyped by BovineHD assay.**

**Table S2. The summary of CNVs or CNVRs for each specie/breed based on Btau_4.0.**

**Table S3. Btau_4.0 CNV regions, their frequencies, corresponding gene contents, QTL, OMIM, and OMIA overlapping information.**

**Table S4. The summary of CNVs or CNVRs for each specie/breed based on UMD3.1.**

**Table S5. UMD3.1 CNV regions, their frequencies, corresponding gene contents.**

**Table S6. The summary of PCR results. See Table S6.xls.**

**Table S7. Over/Underrepresentation of PANTHER terms (molecular function, biological process, pathway, cellular component and PANTHER protein class) on Batu_4.0 and UMD3.1. See Table S7.xls.**

**Table S8. Network, Biological function and Pathway analyses using IPA on Batu_4.0 and UMD3.1. See Table S8.xls.**

Figure S1. Comparison of cattle copy number variations derived from BovineHD and BovineSNP50 assays on Batu_4.0. CNV regions (3,346 events, 143 Mb, ~4.70 % of the bovine genome) derived from BovineHD assay are shown above the chromosomes in green (gain), red (loss) and dark blue (both), while below are the CNV regions (682 events, 139 Mb, ~4.6 % of the bovine genome) derived from BovineSNP50 assay. The bar height represents their frequencies: short (appeared in 1 sample), median (≥2 samples) and tall (≥5 samples). Segmental duplications (94.4 Mb, 3.1% of the bovine genome) predicted by two independent computational approaches are illustrated on the chromosomes in red (WSSD), blue (WGAC) or purple (both). The patterns are depicted for all duplications for ≥5 kb in length and ≥90% sequence identity. The gaps in the assembly are represented on the chromosomes as white ticks.

**Table S1. Numbers of subspecies, breeds, animals and trios used to call CNVs genotyped by BovineHD** assay.

| ***Breed or species*** | ***Acronym*** | ***# Animals*** | ***# Males*** | ***# Females*** | ***# Trios*** |
| --- | --- | --- | --- | --- | --- |
| ***Taurine*** |  |  |  |  |  |
| Holstein | HOL | 66 | 61 | 5 | 5 |
| Limousin | LMS | 48 | 45 | 3 | 1 |
| Angus | ANG | 47 | 43 | 4 | 3 |
| Jersey | JER | 42 | 39 | 3 | 5 |
| Charolais | CHL | 40 | 34 | 6 | 3 |
| Hereford | HFD | 37 | 33 | 4 | 2 |
| Brown Swiss | BSW | 24 | 21 | 3 | 3 |
| Piedmontese | PMT | 24 | 16 | 8 | 3 |
| Ramagnola | RMG | 23 | 20 | 3 | 2 |
| Guernsey | GNS | 21 | 1 | 20 | 1 |
| Norwegian Red | NRC | 14 | 11 | 3 | 0 |
| Wagyu | WAG | 13 | 13 | 0 | 0 |
| Senepol | SEP | 12 | 12 | 0 | 0 |
| Red Angus | RGU | 11 | 10 | 1 | 0 |
| Simmental | SIM | 10 | 10 | 0 | 0 |
| Blonde d'Aquitaine | BDA | 5 | 5 | 0 | 0 |
| Montbeliard | MBL | 5 | 5 | 0 | 0 |
| Normande | NOR | 5 | 4 | 1 | 0 |
|  |  |  |  |  |  |
| ***Indicine*** |  |  |  |  |  |
| Brahman | BRM | 49 | 36 | 13 | 3 |
| Nelore | NEL | 34 | 22 | 12 | 1 |
| Gir | GIR | 30 | 26 | 4 | 3 |
|  |  |  |  |  |  |
| ***Taurine×Indicine*** |  |  |  |  |  |
| Santa Gertrudis | SGT | 35 | 32 | 3 | 3 |
| Beefmaster | BMA | 24 | 23 | 1 | 1 |
| Hybrid Brangus | BRA | 8 | 7 | 1 | 0 |
|  |  |  |  |  |  |
| ***African Breeds*** |  |  |  |  |  |
| N'Dama (Taurine) | NDA | 24 | 4 | 20 | 2 |
| Sheko(Ancient Taurine×Indicine hybrid) | SHK | 18 | 5 | 13 | 0 |
| Lagunaire | LGN | 5 | 0 | 5 | 0 |
|  |  |  |  |  |  |
| **Total** |  | 674 | 538 | 136 | 41 |

**Table S2. The summary of CNVs or CNVRs for each specie/breed based on Btau_4.0.**

| ***Breed or species*** | ***Sample*** | ***Count*** | ***Unique*** | ***Gain*** | ***Loss*** | ***Length*** |
| --- | --- | --- | --- | --- | --- | --- |
| ***Taurine*** |  |  |  |  |  |  |
| Holstein | 66 | 2,939 (44.5) | 81 (1.2) | 1,023 (15.5) | 1,916 (29.0) | 110,517,529 (37,604) |
| Limousin | 48 | 2,359 (49.1) | 187 (3.9) | 988 (20.6) | 1,371 (28.6) | 100,502,786 (42,604) |
| Angus | 47 | 1,804 (38.4) | 66 (1.4) | 611 (13.0) | 1,193 (25.4) | 70,723,694 (39,204) |
| Jersey | 42 | 1,892 (45.0) | 74 (1.8) | 591 (14.1) | 1,301 (31.0) | 60,846,786 (32,160) |
| Charolais | 40 | 2,035 (50.9) | 113 (2.8) | 901 (22.5) | 1,134 (28.4) | 83,409,091 (40,987) |
| Hereford | 37 | 1,468 (39.7) | 48 (1.3) | 654 (17.7) | 814 (22.0) | 60,820,138 (41,431) |
| Brown Swiss | 24 | 1,292 (53.8) | 38 (1.6) | 501 (20.9) | 791 (33.0) | 51,853,239 (40,134) |
| Piedmontese | 24 | 1,196 (49.8) | 96 (4.0) | 591 (24.6) | 605 (25.2) | 50,942,374 (42,594) |
| Ramagnola | 23 | 1,190 (51.7) | 59 (2.6) | 458 (19.9) | 732 (31.8) | 38,317,481 (32,200) |
| Guernsey | 21 | 1,066 (50.8) | 28 (1.3) | 438 (20.9) | 628 (29.9) | 54,602,336 (51,222) |
| Norwegian Red | 14 | 739 (52.8) | 105 (7.5) | 213 (15.2) | 526 (37.6) | 37,460,010 (50,690) |
| Wagyu | 13 | 472 (36.3) | 34 (2.6) | 207 (15.9) | 265 (20.4) | 18,598,598 (39,404) |
| Senepol | 12 | 492 (41.0) | 27 (2.3) | 216 (18.0) | 276 (23.0) | 20,966,465 (42,615) |
| Red Angus | 11 | 421 (38.3) | 20 (1.8) | 154 (14.0) | 267 (24.3) | 17,768,123 (42,205) |
| Simmental | 10 | 315 (31.5) | 19 (1.9) | 126 (12.6) | 189 (18.9) | 12,496,763 (39,672) |
| Blonde d'Aquitaine | 5 | 219 (43.8) | 18 (3.6) | 84 (16.8) | 135 (27.0) | 8,777,260 (40,079) |
| Montbeliard | 5 | 198 (39.6) | 11 (2.2) | 66 (13.2) | 132 (26.4) | 6,990,686 (35,306) |
| Normande | 5 | 205 (41.0) | 20 (4.0) | 94 (18.8) | 111 (22.2) | 8,853,659 (43,189) |
| Subtotal | 447 | 20,302 (45.4) | 1,044 (2.3) | 7,916 (17.7) | 12,386 (27.7) | 814,447,018 (40,117) |
| ***Indicine*** |  |  |  |  |  |  |
| Brahman | 49 | 3,134 (64.0) | 142 (2.9) | 1,232 (25.1) | 1,902 (38.8) | 111,958,673 (35,724) |
| Nelore | 34 | 2,052 (60.4) | 90 (2.6) | 632 (18.6) | 1,420 (41.8) | 77,397,469 (37,718) |
| Gir | 30 | 2,166 (72.2) | 77 (2.6) | 731 (24.4) | 1,435 (47.8) | 77,497,149 (35,779) |
| Subtotal | 113 | 7,352 (65.1) | 309 (2.7) | 2,595 (23.0) | 4,757 (42.1) | 266,853,291 (36,297) |
| ***Taurine×Indicine*** |  |  |  |  |  |  |
| Santa Gertrudis | 35 | 1,832 (52.3) | 70 (2.0) | 791 (22.6) | 1,041 (29.7) | 78,043,729 (42,600) |
| Beefmaster | 24 | 1,377 (57.4) | 93 (3.9) | 585 (24.4) | 792 (33.0) | 63,235,060 (45,922) |
| Hybrid Brangus | 8 | 360 (45.0) | 35 (4.4) | 132 (16.5) | 228 (28.5) | 18,456,981 (51,269) |
| Subtotal | 67 | 3,569 (53.3) | 198 (3.0) | 1,508 (22.5) | 2,061 (30.8) | 159,735,770 (44,756) |
| ***African Breeds*** |  |  |  |  |  |  |
| N'Dama | 24 | 1,691 (70.5) | 112 (4.7) | 687 (28.6) | 1,004 (41.8) | 63,620,438 (37,623) |
| Sheko | 18 | 1,120 (62.2) | 97 (5.4) | 451 (25.1) | 669 (37.2) | 53,538,229 (47,802) |
| Lagunaire | 5 | 277 (55.4) | 31 (6.2) | 110 (22.0) | 167 (33.4) | 12,619,008 (45,556) |
| Subtotal | 47 | 3,088 (65.7) | 240 (5.1) | 1,248 (26.6) | 1,840 (39.1) | 129,777,675 (42,026) |
| **Total** | 674 | 34,311 (50.9) | 1,791 (2.7) | 13,267 (19.7) | 21,044 (31.2) | 1,370,813,754 (39,953) |
| **CNVR** | 674 | 3,346 | 1,316 | 986 | 2,051 | 142,718,107 (42,653) |

**Table S3. Btau_4.0 CNV regions, their frequencies, corresponding gene contents, QTL, OMIM, and OMIA overlapping information. See Table S3.xls.** The description of CNV regions includes the coordinates (chromosome, start position, end position, length, start SNP name, end SNP name, number of encompassing SNPs), CNV type (gain, loss, both), the number of animals having CNV events in this region, as well as its frequency. The frequency was defined as “Unique” when the CNV region was unique only to one animal, “Multiple” when the CNV region was shared by 2 animals, or shown as the exact proportion of animals having CNV events in this region when the CNV region was shared by at least 3 animals. The gene contents of CNV regions include the RefSeq genes, *in silico* mapped human RefSeq genes, Glean consensus genes and Ensembl genes.

**Table S4. The summary of CNVs or CNVRs for each specie/breed based on UMD3.1.**

| ***Breed or species*** | ***Sample*** | ***Count*** | ***Unique*** | ***Gain*** | ***Loss*** | ***Length*** |
| --- | --- | --- | --- | --- | --- | --- |
| ***Taurine*** |  |  |  |  |  |  |
| Holstein | 66 | 4,863(73.7) | 111(1.7) | 1,671(25.3) | 3,192(48.4) | 223,660,576(45,992) |
| Angus | 47 | 3,136(66.7) | 70(1.5) | 997(21.2) | 2,139(45.5) | 139,550,007(44,499) |
| Limousin | 43 | 3,380(78.6) | 99(2.3) | 1,506(35.0) | 1,874(43.6) | 164,344,821(48,623) |
| Jersey | 40 | 2,865(71.6) | 59(1.5) | 992(24.8) | 1,873(46.8) | 127,551,602(44,521) |
| Charolais | 37 | 2,962(80.1) | 127(3.4) | 1,218(32.9) | 1,744(47.1) | 134,993,920(45,575) |
| Hereford | 37 | 2,592(70.1) | 57(1.5) | 1,157(31.3) | 1,435(38.8) | 128,879,067(49,722) |
| Brown Swiss | 24 | 1,994(83.1) | 52(2.2) | 752(31.3) | 1,242(51.8) | 98,506,393(49,401) |
| Piedmontese | 24 | 1,981(82.5) | 110(4.6) | 876(36.5) | 1,105(46.0) | 97,138,179(49,035) |
| Ramagnola | 23 | 1,959(85.2) | 60(2.6) | 791(34.4) | 1,168(50.8) | 86,623,968(44,218) |
| Guernsey | 21 | 1,660(79.0) | 41(2.0) | 591(28.1) | 1,069(50.9) | 99,328,917(59,837) |
| Wagyu | 13 | 891(68.5) | 42(3.2) | 325(25.0) | 566(43.5) | 42,405,446(47,593) |
| Norwegian Red | 12 | 943(78.6) | 87(7.3) | 304(25.3) | 639(53.3) | 53,546,904(56,784) |
| Red Angus | 11 | 798(72.5) | 31(2.8) | 246(22.4) | 552(50.2) | 36,341,005(45,540) |
| Senepol | 11 | 767(69.7) | 27(2.5) | 321(29.2) | 446(40.5) | 36,660,542(47,797) |
| Simmental | 10 | 613(61.3) | 23(2.3) | 244(24.4) | 369(36.9) | 29,358,823(47,894) |
| Blonde d'Aquitaine | 5 | 351(70.2) | 17(3.4) | 144(28.8) | 207(41.4) | 20,633,216(58,784) |
| Montbeliard | 5 | 331(66.2) | 12(2.4) | 129(25.8) | 202(40.4) | 14,012,403(42,334) |
| Normande | 5 | 359(71.8) | 27(5.4) | 155(31.0) | 204(40.8) | 18,088,591(50,386) |
| Subtotal | 434 | 32,445(74.8) | 1,052(2.4) | 12,419(28.6) | 20,026(46.1) | 1,551,624,380(47,823) |
| ***Indicine*** |  |  |  |  |  |  |
| Brahman | 41 | 3,276(79.9) | 166(4.0) | 1,342(32.7) | 1,934(47.2) | 165,024,156(50,374) |
| Nelore | 30 | 2,654(88.5) | 89(3.0) | 778(25.9) | 1,876(62.5) | 112,300,261(42,314) |
| Gir | 26 | 2,785(107.1) | 72(2.8) | 982(37.8) | 1,803(69.3) | 117,641,875(42,241) |
| Subtotal | 97 | 8,715(89.8) | 327(3.4) | 3,102(32.0) | 5,613(57.9) | 394,966,292(45,320) |
| ***Taurine×Indicine*** |  |  |  |  |  |  |
| Santa Gertrudis | 35 | 2,962(84.6) | 92(2.6) | 1,277(36.5) | 1,685(48.1) | 137,852,841(46,540) |
| Beefmaster | 20 | 1,778(88.9) | 105(5.3) | 795(39.8) | 983(49.2) | 86,541,927(48,674) |
| Hybrid Brangus | 8 | 592(74.0) | 40(5.0) | 196(24.5) | 396(49.5) | 32,354,414(54,653) |
| Subtotal | 63 | 5,332(84.6) | 237(3.8) | 2,268(36.0) | 3,064(48.6) | 256,749,182(48,153) |
| ***African Breeds*** |  |  |  |  |  |  |
| N'Dama | 19 | 1,784(93.9) | 109(5.7) | 662(34.8) | 1,122(59.1) | 75,701,691(42,434) |
| Sheko | 12 | 1,039(86.6) | 83(6.9) | 403(33.6) | 636(53.0) | 54,032,717(52,005) |
| Lagunaire | 5 | 389(77.8) | 38(7.6) | 132(26.4) | 257(51.4) | 21,825,356(56,106) |
| Subtotal | 36 | 3,212(89.2) | 230(6.4) | 1,197(33.3) | 2,015(56.0) | 151,559,764(47,185) |
| **Total** | 630 | 49,704(78.9) | 1,846(2.9) | 18,986(30.1) | 30,718(48.8) | 2,354,899,618(47,378) |
| **CNVR** | 630 | 3,438 | 1,360 | 1,054 | 2,042 | 146,905,950(42,730) |

**Table S5. UMD3.1 CNV regions, their frequencies, corresponding gene contents. See Table S5.xls.** The description of CNV regions includes the coordinates (chromosome, start position, end position, length, start SNP name, end SNP name, number of encompassing SNPs), CNV type (gain, loss, both), the number of animals having CNV events in this region, as well as its frequency. The frequency was defined as “Unique” when the CNV region was unique only to one animal, “Multiple” when the CNV region was shared by 2 animals, or shown as the exact proportion of animals having CNV events in this region when the CNV region was shared by at least 3 animals.

**Table S6. The summary of PCR results. See Table S6.xls.**

**Table S7. Over/Underrepresentation of PANTHER terms (molecular function, biological process, pathway, cellular component and PANTHER protein class) on Batu_4.0 and UMD3.1. See Table S7.xls.**

**Table S8. Network, Biological function and Pathway analyses using IPA on Batu_4.0 and UMD3.1. See Table S8.xls.**

Figure S1. Comparison of cattle copy number variations derived from BovineHD and BovineSNP50 assays on Batu_4.0.
