# Supplementary figures and images for: Fine mapping of copy number variations on two cattle genome assemblies using high density SNP array
Source: BMC Genomics. 2012 Aug 6;13:376. doi: 10.1186/1471-2164-13-376 (PMC3583728; doi:10.1186/1471-2164-13-376)

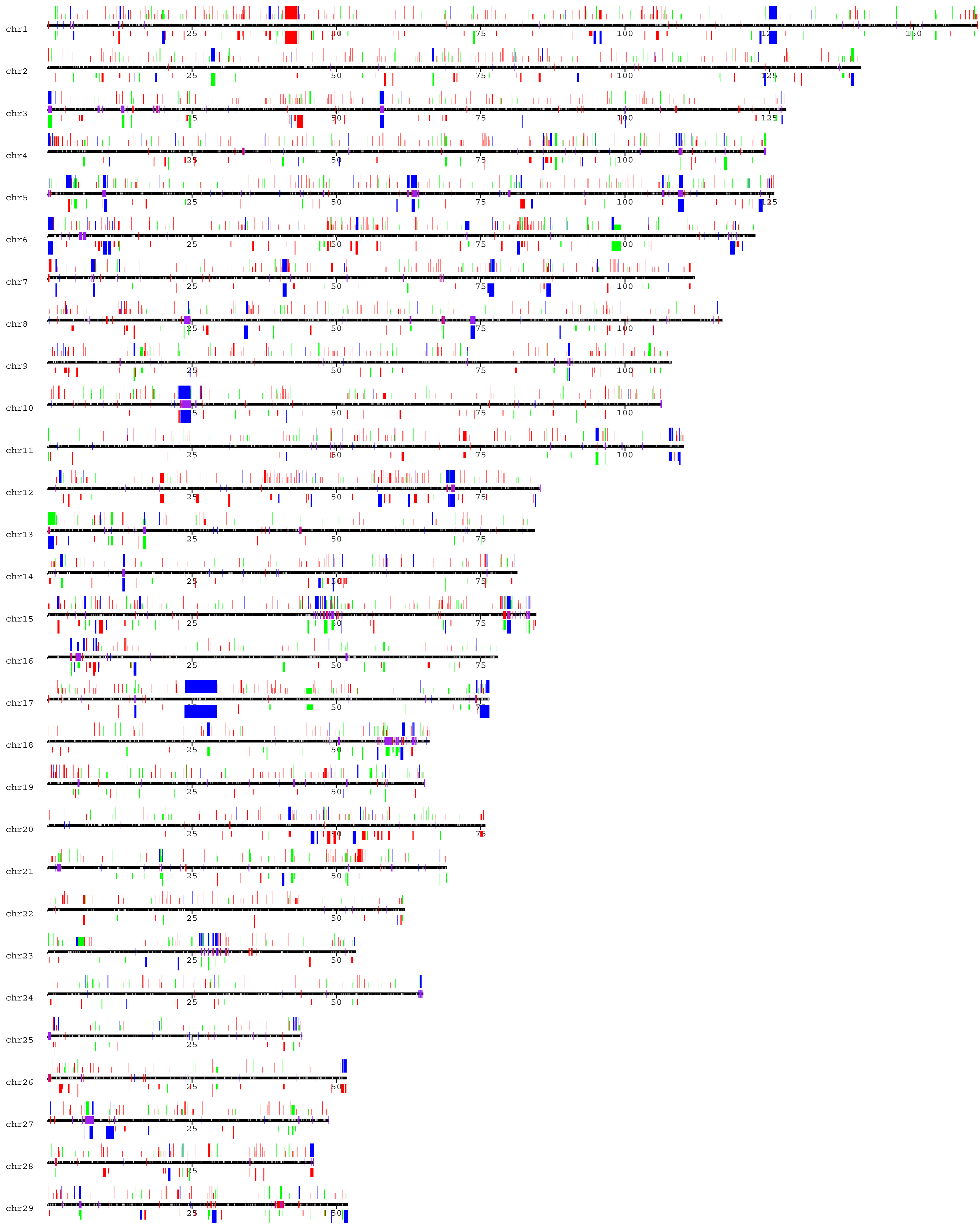

Supplement: Additional file 7 — Table S8: Network, Biological function and Pathway analyses using IPA on Batu_4.0 and UMD3.1. [file 1471-2164-13-376-S7.pdf]
